# Enhanced Impact Resistance, Oxygen Barrier, and Thermal Dimensional Stability of Biaxially Processed Miscible Poly(Lactic Acid)/Poly(Butylene Succinate) Thin Films

**DOI:** 10.3390/polym16213033

**Published:** 2024-10-29

**Authors:** Piyawanee Jariyasakoolroj, Pramote Kumsang, Supanut Phattarateera, Noppadon Kerddonfag

**Affiliations:** 1 Department of Packaging and Materials Technology, Faculty of Agro-Industry, Kasetsart University, Bangkok 10900, Thailand; 2 Center for Advanced Studies for Agriculture and Food (CASAF), Kasetsart University, Bangkok 10900, Thailand; 3 National Metal and Materials Technology Center, National Science and Technology Development Agency (NSTDA), Ministry of Higher Education, Science, Research and Innovation, Khlong Luang 12120, Pathum Thani, Thailand

**Keywords:** polymer blends, poly(lactic acid), poly(butylene succinate), biaxial stretching, impact strength, film shrinkage

## Abstract

This study investigates the crystallization, microstructure, and performance of poly(lactic acid)/poly(butylene succinate) (PLA/PBS) thin films processed through blown film extrusion and biaxial orientation (BO) at various blend ratios. Succinic anhydride (SA) was used to enhance interfacial adhesion in PLA-rich blends, while blends near 50/50 formed co-continuous phases without SA. Biaxial stretching and annealing, adjusted according to the crystallization behavior of PLA and PBS, significantly influenced crystallinity, crystallite size, and molecular orientation. Biaxial stretching induced crystallization and ordered chain alignment, particularly at the cold crystallization temperature (T_cc_), leading to a 70–80-fold increase in impact resistance compared to blown films. Annealing further enhanced crystallinity, especially at the T_cc_ of PLA, resulting in larger crystallite sizes. BO films demonstrated reduced thermal shrinkage due to improved PLA crystalline structure, whereas PLA-rich blown films showed higher shrinkage due to PLA’s lower thermal resistance. The SA-miscibilized phase reduced oxygen transmission in blown films, while BO films exhibited higher permeability due to anisotropic crystal orientation. However, the annealing of BO films, especially at high temperature (T_cc_ of PLA), further lowered oxygen permeability by promoting the crystallization of both PLA and PBS phases. Overall, the combination of SA compatibilization, biaxial stretching, and annealing resulted in substantial improvements in mechanical strength, dimensional stability, and oxygen barrier properties, highlighting the potential of these films for packaging applications.

## 1. Introduction

Addressing the substantive environmental concerns related to plastic pollution and the consequential carbon emissions contributing to global warming, it is noteworthy that biodegradable polymers, such as poly(lactic acid) (PLA), polyhydroxyalkanoates (PHAs), polysaccharide derivatives, poly(ε-caprolactone) (PCL), poly(butylene succinate) (PBS), poly(butylene adipate-co-terephthalate) (PBAT), etc., stand as empirically supported alternatives to conventional plastics [1,2].

Among these, PLA stands out as a reliable choice for industrial-scale production, utilizing plant-based resources. It has appealing characteristics, including high transparency, a glossy finish, low sealing temperature, moderate gas permeation, and compliance with safety standards for food contact materials. These properties make PLA a compelling option for manufacturing disposable items, particularly in food packaging applications such as lid films, wraps, and stretch films, which are traditionally produced from polyethylene terephthalate (PET) [1,3,4]. Despite these advantages, the brittleness and low thermal stability of PLA limit its broader application. To overcome these challenges, a strategic approach involves physically blending PLA with flexible biodegradable polymers to increase crystallinity, thereby enhancing toughness and thermal resistance. For instance, the incorporation of PCL, a biocompatible and biodegradable polymer, into PLA matrices has been extensively reported to enhance PLA crystallinity, resulting in improved toughness [5,6,7]. However, the use of PCL is limited by its petroleum-based origin and high cost. On the other hand, blending PLA with PBAT has been found to increase the ductility and impact resistance of PLA-based blends. Nevertheless, PBAT slowed the degradation rate, lowered the thermal resistance, and exhibited compatibility issues with PLA [8,9,10]. In this regard, PBS emerges as a promising alternative, not only for enhancing the toughness of PLA but also for improving its thermal dimensional stability [11,12]. Additionally, PBS can be produced from renewable resources, offering advantages such as melt processability, a high crystallization rate, compostability, and flexibility [13,14].

However, several studies have indicated a notable phase separation between PLA and PBS. For example, Yokohara and Yamaguchi conducted a morphological study of PLA/PBS composite materials using a scanning electron microscope (SEM) [15]. Wu et al. observed phase separation between PLA and PBS in their morphological study, where clusters of PBS particles were found adhering to the surface of PLA due to polarity differences, leading to inadequate interfacial adhesion [16]. Additionally, Choi et al. demonstrated that the phase separation occurred in PLA/PBS blend fibers with high interfacial tension, particularly at PBS contents exceeding 10 wt.%, owing to thermodynamic incompatibility [17].

To date, several methods have been investigated to enhance the compatibility of PLA/PBS blends, including the incorporation of reactive compatibilizers, copolymerization, the utilization of nanoparticles or nanofillers, and the optimization of blending ratios [18]. Among these approaches, reactive compatibilizers have emerged as particularly promising strategies, offering versatility and long-term stability in improving interfacial adhesion through the formation of covalent bonds [19,20]. In essence, the use of reactive compatibilizers can facilitate in situ copolymerization, leading to decreased interfacial tension and improved compatibility [21,22,23]. These compatibilizers typically consist of various multi-functional groups, such as diisocyanate [24,25,26], epoxy groups [27,28,29], free radical initiators [30,31,32], etc., which react with the carboxyl and hydroxyl groups present in PLA and PBS chains. Considering factors such as chemical reactivity, versatility, safety for food contact, and cost-effectiveness, our recent study investigated the application of succinic anhydride (SA) as a reactive compatibilizer to effectively enhance the miscibility and thermal dimensional stability of PLA/PBS thin films [33].

In recent decades, while reactive PLA-based blending has been extensively investigated to enhance PLA crystallization, the biaxial stretching process, or biaxial orientation (BO), has also emerged as another effective method to improve PLA crystallization. Numerous studies have reported on the microstructural characteristics and related properties of biaxially oriented PLA (BOPLA), including improvements in toughness, thermal behavior, and thermal dimensional stability [34,35,36,37]. Additionally, the BO process has been applied to PLA-based blends. For example, Al-try et al. studied the crystallization and thermal properties of BO-PLA/PBAT with/without multi-functional styrene–acrylic oligomers (Joncryl^®^ ADR-4368 (BASF, Ludwigshafen, Germany)) as compatibilizers, stretching them above the glass transition temperature. Their results showed that Joncryl accelerated PLA crystallization and maintained this effect in PLA/PBAT blends without affecting PBAT crystallization [38]. Liu et al. investigated BO-PLA/PBAT films, finding that stretching increased PLA crystallinity from 6% to 58.6% and improved oxygen barrier properties by 22%. The stretched films also showed enhanced tensile strength and surface roughness variations, making them highly suitable for packaging applications [39]. While existing studies have primarily focused on BO-PLA/PBAT blends, where PLA and PBAT form a predominantly amorphous/amorphous binary polymer blend system, research on the BO process applied to PLA blended with highly crystalline polymers such as PBS is limited. Furthermore, the effects of temperature conditions during the BO process remain underexplored.

In the study, the weight ratios of PLA/PBS blends were varied, and SA was employed as a reactive compatibilizer to induce the formation of an SA-miscibilized PLA/PBS region within the system. The BO process, with varying stretching and annealing temperatures, was applied to PLA/PBS and PLA/PBA/SA films. Additionally, PLA/PBS and PLA/PBS/SA thin films, produced via blown film extrusion—a process that imparts a biaxial orientation through the bubbling stage—were investigated to compare chain orientation and material properties. A comprehensive analysis was performed to examine the correlation between microstructure and the thermal and mechanical properties of biaxially processed PLA/PBS thin films, with and without the incorporation of SA, to evaluate their potential in food packaging applications.

## 2. Materials and Methods

### 2.1. Materials

PLA (Ingeo^TM^ Biopolymer 4043D) with a melt flow rate (MFR) of 5.45 g/10 min, a density of 1.24 g/cm^3^, and number-averaged molecular weight (*M_n_*) and weight-averaged molecular weight (*M_w_*) of 110 kDa and 170 kDa, respectively, was obtained from NatureWorks^®^ LLC, Plymouth, MN, USA. PBS (BioPBS™ FZ91PM) with an MFR of 5.52 g/10 min, a density of 1.26 g/cm^3^, and *M_n_* and *M_w_* of 48 kDa and 150 kDa, respectively, was sourced from PTT MCC Biochem Co., Ltd., Rayong, Thailand. Succinic anhydride (SA) with a purity of ≥99% (GC) and commercial code 239690 was obtained from Merck Pte. Ltd., Singapore.

### 2.2. Preparation of Biaxially Processed PLA/PBS and PLA/PBS/SA Thin Films

**Scheme 1:**
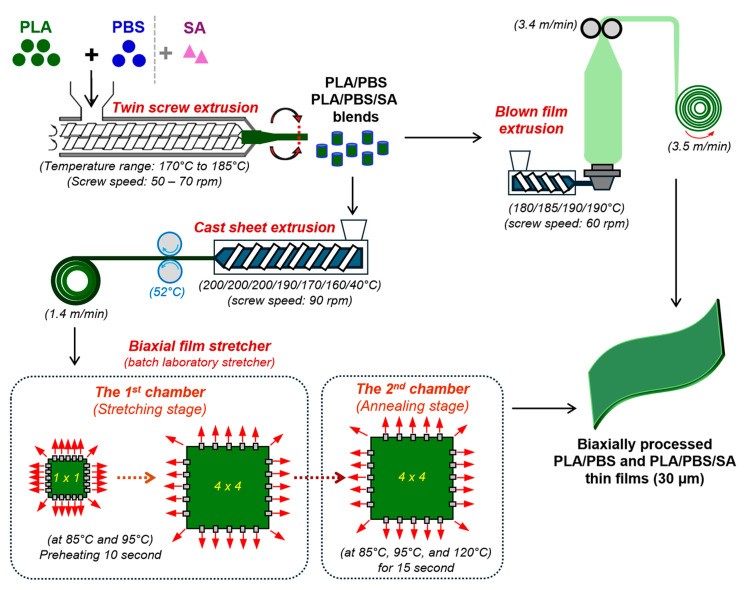
Biaxially processed PLA/PBS and PLA/PBS/SA thin film preparation.

PLA and PBS resins were initially dry mixed at weight ratios of 80/20, 70/30, and 60/40 using a Siemens SIMATIC HMI high-speed mixer. Additionally, 0.5 phr SA was incorporated into the dry blends, based on the optimal conditions established in our previous study [27]. The mixtures were subsequently melt blended using a Labtech Engineering LTE 20–40 twin screw extruder (Labtech Engineering Co., Ltd., Samut Prakan, Thailand), with processing temperatures ranging from 170 °C to 185 °C and screw speeds between 50 and 70 rpm. The resulting PLA/PBS and PLA/PBS/SA blends were processed into blown thin films with a final thickness of 30 μm using a Labtech Engineering LE 20–30 single-screw extruder, equipped with a Labtech Engineering LF 400 blown film unit (Labtech Engineering Co., Ltd., Samut Prakan, Thailand), as summarized in Table 1. The temperature profile from the feed zone to the die zone was maintained at 180/185/190/190 °C. The rotational speeds of the single-screw, nip rolls, and take-up were set to 60 rpm, 3.4 m/min, and 3.5 m/min, respectively, as shown in Scheme 1.

In addition, precursor sheets of PLA/PBS and PLA/PBS/SA blends, with a thickness of 300–330 μm, were fabricated using a Collin Teach-Line^®^ E20 TH single-screw extruder (COLLIN Lab & Pilot Solutions GmbH, Maitenbeth, Germany). This laboratory-scale flat die film line featured a 20 mm diameter extruder with an L/D ratio of 25, a 2.5D mixing section at the end, and a compression ratio of 3:1. A 200 mm wide T-die with a 0.3 mm gap was used. The temperatures of the barrel (zones 1–4), adapter (zone 5), and die (zones 6–7) were set to 40 °C, 160 °C, 170 °C, 190 °C, 200 °C, 200 °C, and 200 °C, respectively. Sheets were extruded at a screw speed of 90 rpm and quenched on a water-cooled chill roll at 52 °C, with a take-up speed of 1.4 m/min. Using a Brückner Karo IV batch laboratory stretcher (Brückner Group SE, Siegsdorf, Germany), the sheets were preheated to the stretching temperature for 10 s, followed by biaxial stretching at 85 °C and 95 °C in the first chamber. The sheets were simultaneously stretched in both the machine and transverse directions (MD and TD) with a 4 × 4 draw ratio. The resulting films, with a thickness of 30 μm, were then annealed in the s chamber with uniform heating by air circulation at 85 °C, 95 °C, and 120 °C for 15 s, as detailed in Table 1.

### 2.3. Characterization

The PLA/PBS and PLA/PBA/SA blends were prepared via extrusion, and the extrudate was pelletized to obtain resin samples. These pelletized samples were cryo-fractured to expose the cross-sectional surface and examined using a FEI Quanta 450 SEM (FEI Technologies Inc., Hillsboro, OR, USA), after gold sputter coating for 2 min, at 8000× magnification.

Thermal properties of the samples were investigated using a METTLER TOLEDO STAR^e^ System differential scanning calorimeter (DSC) (Mettler-Toledo (Switzerland) GmbH, Greifensee, Switzerland). A 20 μL aluminum pan with a lid, recommended for thin films, was used. The sample was heated from 25 °C to 200 °C at a rate of 30 °C/min under a nitrogen flow rate of 50 mL/min. The degree of crystallinity (*X_c_*) was calculated using the following equation:

*X_c_* (%) = [(ΔH_m_ − ΔH_cc_) × 100%] × (*w* × ΔH^0^_m_)^−1^ (1)

where ΔH_m_ and ΔH_cc_ are the melting and cold crystallization enthalpies, and ΔH^0^_m_ is the heat fusion for a 100% crystalline polymer (93.1 J/g for PLA [40] and 110.3 J/g for PBS [41], respectively). The variable *w* represents the weight fraction of PLA and PBS.

The PLA/PBS and PLA/PBS/SA precursor sheets and films were cut into pieces measuring 2 cm in length and 1 cm in width. The 2D-WAXD measurements were conducted at Beamline BL1.3W at the Siam Photon Laboratory of the Synchrotron Light Research Institute (SLRI), Nakhon Ratchasima, Thailand. The X-ray beam was generated with an energy of 9 keV and a wavelength of 1.38 Å. Data collection was performed using a Rayonix LX170HS detector with an exposure time of 420 s. The detector was positioned 163.82 mm from the sample. The WAXD patterns were analyzed using an in-house developed Matlab-based data-processing program called SAXSIT (V.4.47). The 2D-WAXD patterns were integrated along the equatorial direction to obtain 1D-WAXD profiles, and the crystallite size (<L>) was calculated using the Scherrer equation [42]:

<L> = (*K* × λ) × (FWHM × cos *θ*)^−1^ (2)

where *K* is the shape factor, typically 0.9, FWHM is the full width at half maximum of the identified diffraction peak (in radians), and *θ* is the Bragg angle (in degrees).

The impact resistance of thin film samples was evaluated using a Labthink FIT-01 film pendulum impact tester (Labthink Instruments Co., Ltd., Jinan, China), following ASTM D3420-21 (2021). Films were cut into square specimens and tested with a 1 Joule load cell. The impact strength was calculated based on the energy absorbed during fracture and the cross-sectional area of the film. A minimum of five specimens were tested for each sample type, with results reported as the average impact strength and corresponding standard deviation.

The oxygen permeability of the thin film samples, with a thickness of approximately 30 μm, was measured using a Systech Illinois 8101e OxySense^®^ oxygen transmission rate analyzer (Illinois Instruments Inc, Johnsburg, IL, USA) in accordance with ASTM D3985-17 (2017). Prior to testing, the films were conditioned at 25 °C and 50% relative humidity for 48 h. The oxygen transmission rate (OTR) was determined by quantifying the amount of oxygen permeating through the film over a specific time interval. The results were expressed in units of cc.mm/m^2^.day.atm, calculated by multiplying the measured OTR value (cc/m^2^.day.atm) by the film thickness (mm).

Heat shrinkage of the thin film samples was measured using an adapted method from ASTM D1204-14 (2020). The films were cut into 50 mm × 50 mm specimens, and initial dimensions were recorded. The specimens were placed in a preheated oven at 120 °C for 60 min. After the heating period, the samples were removed and allowed to cool to room temperature. The final dimensions were measured, and the heat shrinkage percentage was calculated as follows:

Heat shrinkage (%) = [(Initial length − Final length) × 100%] × (Initial length)^−1^ (3)

The shrinkage was evaluated in both the MD and TD. The results were reported as the average shrinkage percentage from a minimum of five specimens per film type.

## 3. Results and Discussion

### 3.1. Morphological Observation of PLA/PBS and PLA/PBS/SA Blends

**Figure 1:**
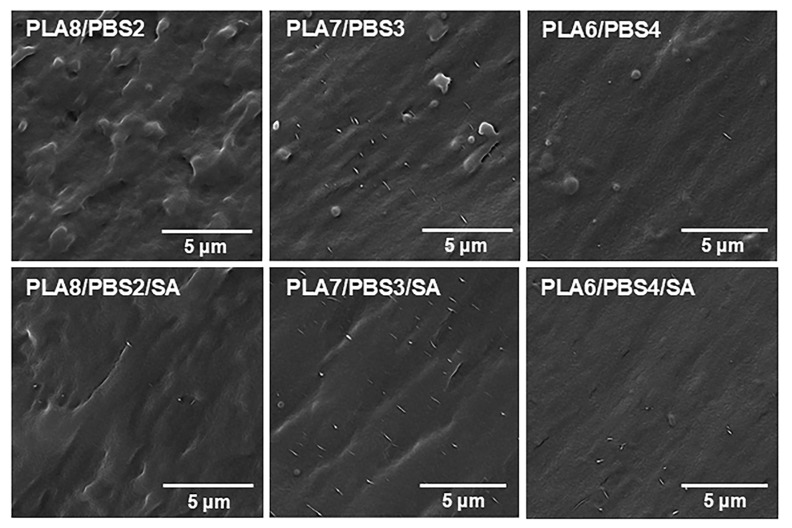
Cross-sectional SEM micrographs of PLA/PBS and PLA/PBS/SA blends at different weight ratios.

The cross-sectional surface morphologies of PLA/PBS blends at various weight ratios were examined using SEM, as shown in Figure 1. PLA8/PBS2 and PLA7/PBS3 exhibited rough surfaces, while PLA6/PBS4, with a 40 wt.% PBS content, displayed a smoother surface. This aligns with the findings of Wu et al. and Deng et al., who reported co-continuous phase formation in PLA/PBS blends at PBS contents of approximately 40–50 wt.% [16,43].

Incorporating SA to improve interfacial adhesion between the PLA and PBS phases resulted in smoother surfaces with fewer PBS droplets in the PLA/PBS/SA samples. This suggests that SA chemically interacted with both PLA and PBS, enhancing miscibility and forming SA-miscibilized regions within the blend, consistent with our previous findings. As described in our prior work, SA was hydrolyzed to succinic acid, and the dicarboxylic acid groups underwent esterification with hydroxyl groups at the chain ends of PLA and PBS. X-ray photoelectron spectroscopy (XPS) confirmed this reaction, showing an increased ester-to-hydrocarbon (COO/C) ratio with rising SA content in the PLA/PBS system [33]. The formation of the SA-miscibilized PLA/PBS regions exhibits structural similarities to both the PLA and PBS phases, leading to stronger interfacial adhesion and a more homogeneous blend. These results align with prior studies on miscible PLA/PBS blends incorporating random poly(butylene succinate-co-lactic acid) copolymers, which also demonstrated improved phase compatibility and uniform morphology [44].

### 3.2. Thermal Properties and Crystallization of PLA/PBS and PLA/PBS/SA Blends

**Figure 2:**
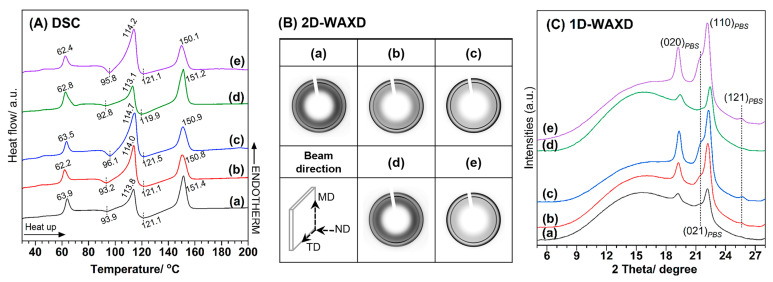
(**A**) First heating DSC thermograms, (**B**) 2D-WAXD patterns, and (**C**) integrated 1D-WAXD profiles for (a) PLA8/PBS2, (b) PLA7/PBS3, (c) PLA6/PBS4, (d) PLA8/PBS2/SA, and (e) PLA6/PBS4/SA blends.

The thermal properties of the PLA/PBS and PLA/PBS/SA blends were examined using DSC technique, as shown in Figure 2A. The first heating DSC scans show that the PLA/PBS blends displayed endothermic peaks at 62–64 °C, ~114 °C, and ~150 °C, corresponding to the mesophase of PLA, melting of PBS (T_m,PBS_), and melting of PLA (T_m,PLA_), respectively. The calculated crystallinity of the PBS phase (*X_c,PBS_*) remained around 40% despite the increase in PBS content from 20 wt.% to 40 wt.%, as shown in Table 2. However, the crystallinity of PLA (*X_c,PLA_*) increased from 7.7% to 13.3% with the increased PBS content, suggesting that the rapid crystallization of PBS acted as a nucleating agent, promoting the crystallization of the PLA phase, as reported by Zhang et al. [45] and Shi et al. [46].

Compared to the thermal properties of PLA/PBS and PLA/PBS/SA blends, no significant changes were observed in the endothermic peaks of the miscible PLA/PBS/SA blends. The cold crystallization temperatures of the PBS (T_cc,PBS_) and PLA (T_cc,PLA_) phases in both blends were approximately 93–95 °C and 120 °C, respectively. The *X_c_* values of PLA in the PLA8/PBS2/SA and PLA6/PBS4/SA blends remained comparable to those in the PLA8/PBS2 and PLA6/PBS4 blends. However, *X_c,PBS_* slightly decreased to 36.8% and 39.4% in the PLA8/PBS2/SA and PLA6/PBS4/SA blends, respectively, relative to the PLA8/PBS2 and PLA6/PBS4 systems. This decrease could be attributed to the presence of the SA-miscibilized region within the PLA/PBS phases, which incorporates a PLA segment with a lower crystallization rate, thereby hindering the rapid crystallization of the PBS phase. These observations are consistent with previous findings reported by Tan et al. [47] and Zhang et al. [48].

The crystalline phases of PLA and PBS were analyzed using the 2D-WAXD technique. Figure 2B shows two distinct concentric rings corresponding to the (020), (110), and (121) diffraction planes of PBS crystals at 19.2°, 22.2°, and 25.5° 2*θ*, respectively, with no detectable diffraction planes for PLA [49,50,51]. This absence of PLA diffraction planes indicates its low crystallization rate, resulting in a low *X_c,PLA_* and the lack of clear diffraction features for PLA crystals. In contrast, PBS exhibits a higher crystallization rate, as evidenced by its well-defined diffraction planes. Additionally, the (021) diffraction peak of PBS crystals, which overlaps with the (110) peak, appears as a shoulder, indicating the presence of the *α*-form of the PBS crystal, typically observed in PBS melt blend samples. This observation is consistent with the findings of Wang et al. [52] and our previous study [53].

Furthermore, the diffraction intensities were integrated over a 15° angular sector centered around the hk0 equatorial direction of the sample planes to obtain 1D-WAXD profiles, as shown in Figure 2C. The overall crystallinity (*X_c,WAXD_*) and the crystallite size (<L>) of the (020), (021), and (110) diffraction planes of PBS crystals were quantified using the Scherrer equation, as shown in Table 2. *X_c,WAXD_* of the PLA/PBS and PLA/PBS/SA blends increased with higher PBS content, and a slight increase in <L>. These findings from both DSC and WAXD analyses suggest that the higher proportion of the rapidly crystallizing PBS phase predominantly promotes PBS crystal growth. This behavior reflects the inherently high crystallinity of PBS, similar to observations in crystalline/crystalline blend systems such as PBS/poly(butylene adipate) (PBS/PBA). For example, at high PBS contents, large PBS spherulites occupied the entire area, while PBA lamellae were inserted within the PBS interlamellar regions [54].

### 3.3. Thermal Properties and Crystallization of Biaxially Processed PLA/PBS and PLA/PBS/SA Thin Films

#### 3.3.1. No Annealing Condition

To explore the effects of biaxial stretching on both PLA- and PBS-dominant blends under different conditions, the weight ratios of 80/20 and 60/40 were selected to produce BO-PLA/PBS and BO-PLA/PBS/SA films using biaxial stretching at various stretching and annealing temperatures (Table 1). The T_cc,PBS_ at 95 °C during the first heating stage and the crystallization temperature (T_c,PBS_) at 85 °C during the cooling stages were used to investigate the effects of stretching conditions on microstructural changes and properties (see Figure S1). Additionally, blown films were produced with partial biaxial chain alignment during the bubbling process, enabling a comparative analysis of the influence of chain orientation on microstructure and properties.

**Figure 3:**
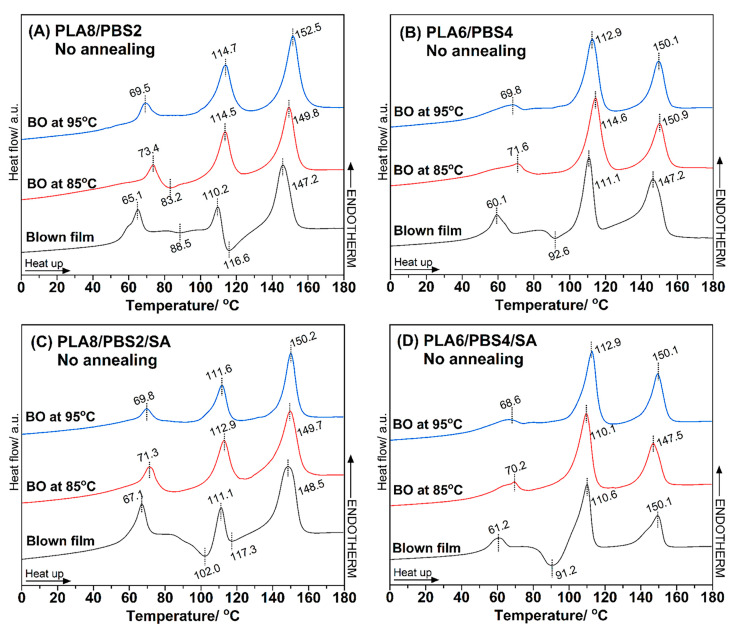
First heating DSC thermograms of blown and BO thin films without annealing for PLA/PBS and PLA/PBS/SA blends.

Figure 3 shows the first heating DSC scans of PLA/PBS and PLA/PBS/SA thin films, produced by blown film extrusion and biaxial stretching without annealing. The blown PLA8/PBS2 film exhibited a mesophase endotherm at 65.1 °C, T_m,PBS_ at 110.2 °C, and T_m,PLA_ at 147.2 °C. Additionally, its exotherm T_cc,PLA_ was observed at 116.6 °C. In contrast, the blown PLA6/PBS4 film showed a mesophase endotherm at 60.1 °C, with the disappearance of T_cc,PLA_. From Table 3, X_c,PLA_ in the blown PLA/PBS films increased with higher PBS content, indicating that PBS served as a nucleating agent for PLA phase. A similar trend was observed in the blown PLA/PBS/SA films with varying PBS content. Concerning strain-induced crystallization, *X_c,PLA_* in the blown PLA/PBS films significantly increased compared to the PLA/PBS melt blends, while *X_c,PBS_* decreased. This suggests that the stretching forces during the bubbling stage effectively promoted ordered chain alignment, particularly in polymers with a low crystallization rate. In contrast, the crystal growth of the rapidly crystallizing PBS phase, dispersed in the blown PLA8/PBS2 film, was hindered by the strain-induced crystallization of the PLA phase. However, the incorporation of SA-miscibilized region-enhancing interfacial adhesion facilitated PBS crystallization in the blown PLA8/PBS2/SA film, with *X_c,PBS_* at 23.6%. This is consistent with previous findings in PLA/PBS blends, as reported by Pivsa-Art et al. [55], Tan and Qu [56], and Kadowaki and Kojio [57].

When the samples were biaxially stretched at 85 °C, neither T_cc,PBS_ nor T_cc,PLA_ were observed, except for the BO-PLA8/PBS2-85 sample, which showed T_cc,PBS_ at 83.2 °C. The enthalpy associated with the PLA mesophase (ΔH_meso_) decreased in comparison to the unstretched blends and blown films, suggesting strain-induced crystallization of both PLA and PBS phases. This observation indicates that biaxial stretching is sufficient to induce ordered chain alignment, unlike the air-induced inflation forces applied during blown film extrusion, as similarly observed in both biaxially oriented polyolefins [58], and biodegradable polyesters [34,35,53,59].

For BO-PLA8/PBS2 and BO-PLA8/PBS2/SA films, which have a PLA-rich phase, the *X_c,PLA_* values exceeded 30% in the presence of an SA-miscibilized PLA/PBS region, as shown in Table 3. In contrast, the *X_c,PLA_* values for BO-PLA6/PBS4 and BO-PLA6/PBS4/SA films, which have a higher PBS content and improved interfacial adhesion, remained stable at 30–35%. This indicates that the enhanced interfacial adhesion between PLA and PBS phases plays a more crucial role in promoting the chain mobility required for the complete crystallization of the slow-crystallizing PLA phase compared to the effects of strain-induced crystallization. These findings align with reports on reactive PLA/PBS blends, as extensively studied by Di Lorenzo [13], Choi et al. [17], Gu et al. [20], and Hao et al. [24].

Upon comparing the stretching temperatures of 85 °C and 95 °C, the characteristic melting peaks of PLA and PBS are similar. Notably, *X_c,PBS_* of both BO-PLA/PBS and BO-PLA/PBS/SA films stretched at 95 °C increased drastically to 55–65%. This behavior can be attributed to the inherently higher crystallization rate of PBS, as stretching at T_cc,PBS_—a temperature that is high for PBS but low for PLA relative to their respective melting temperatures—preferentially promotes chain mobility in the PBS phase. This conclusion is supported by studies demonstrating that PBS undergoes recrystallization at elevated temperatures near its melting point, leading to the formation of stable crystalline structures [50,51].

**Figure 4:**
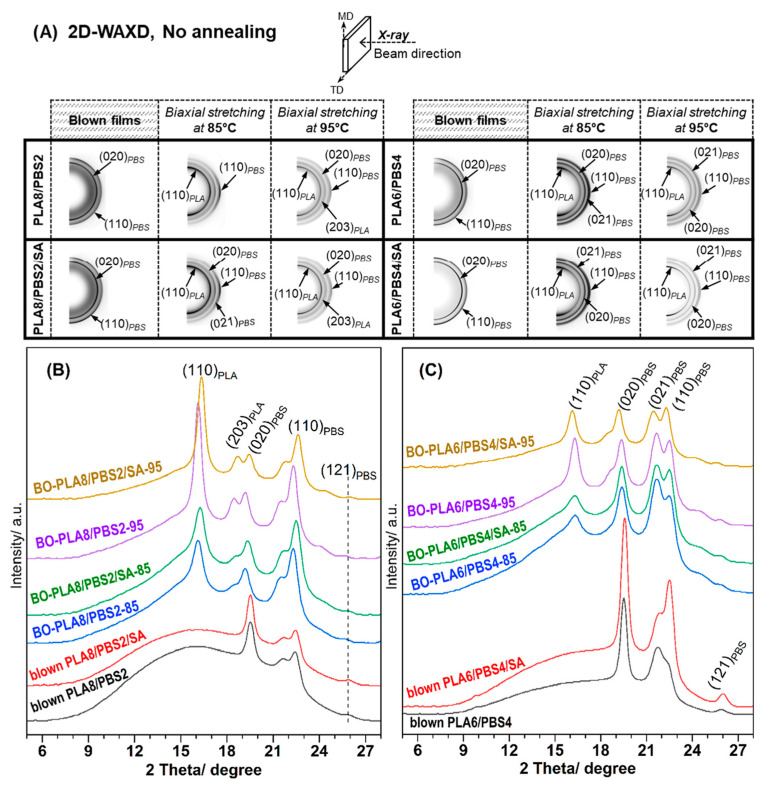
(**A**) 2D-WAXD and (**B**,**C**) 1D-WAXD patterns of blown and BO films without annealing for PLA/PBS and PLA/PBS/SA systems.

In the 2D-WAXD analysis of blown PLA/PBS and PLA/PBS/SA films, the diffraction patterns of the PBS (020) and (110) planes closely resemble those of their respective blends, with no detectable PLA crystalline diffraction patterns, as shown in Figure 4A. Conversely, the (110) diffraction plane of the PLA crystal is clearly observed in BO-PLA/PBS and BO-PLA/PBS/SA films, along with the PBS (020) and (110) diffraction planes. Additionally, at higher stretching temperatures (95 °C), the (203) diffraction plane of the PLA crystal becomes apparent in the BO-PLA8/PBS2-95 and BO-PLA8/PBS2/SA-95 films.

Comparing the 1D-WAXD diffraction patterns of BO-PLA/PBS films with the BO-PLA/PBS/SA films at the same blending weight ratios (Figure 4B,C) reveals similar patterns. This similarity indicates that SA-miscibilized PLA/PBS region does not significantly alter the crystalline forms of PLA (*δ*-form) and PBS (*α*-form). However, increasing the PBS content in the PLA/PBS system directly affects the sharpness and intensities of both PLA and PBS diffraction planes. Furthermore, the increased PBS content influences the orientation of the crystalline PLA phase. In the BO-PLA8/PBS2 and BO-PLA8/PBS2/SA films, which contain a PLA-rich phase, the (110) diffraction plane of the PLA crystal exhibits isotropic orientation, irrespective of the stretching temperatures (85 °C and 95 °C), while the PBS crystals predominantly align along the MD. In contrast, the BO-PLA6/PBS4 and BO-PLA6/PBS4/SA films, with higher PBS content, display anisotropic orientation of the (110) PLA diffraction plane, which preferentially aligns along the TD. Additionally, the isotropic orientation of the (021) diffraction plane of the PBS crystalline phase is clearly present in these samples. This behavior can be attributed to the distinct inherent crystallization characteristics of PLA and PBS. In a PLA-rich phase, PLA crystals respond more rapidly to stretching than PBS crystals. Conversely, in systems with high PBS content and co-continuous PLA/PBS phases, the rapid crystallization of PBS, coupled with biaxial stretching disrupts the orientation of PLA crystals. These findings concerning the influence of PLA weight fraction on crystal orientation align with the previous study on stretched PBAT/PLA samples [60].

*X_c,WAXD_* of BO-PLA8/PBS2 and BO-PLA8/PBS2/SA films was significantly higher compared to that of the blown films, with an increase in <L>_(110)PLA_ and <L>_(203)PLA_ to 12–14 nm, corresponding to PLA crystallization induced by biaxial stretching, as typically observed in BOPLA films [35,39]. Moreover, <L>_(110)PLA_ and <L>_(203)PLA_ of BO-PLA8/PBS2-95 film increased to 20 nm and 25 nm, respectively, with increasing stretching temperature, suggesting that stretching at elevated temperatures near T_cc,PLA_ promotes PLA crystallization, in agreement with previous studies [34,61].

For PLA6/PBS4 and PLA6/PBS4/SA films, which contain a high content of rapidly crystallizing PBS, *X_c,WAXD_* and the PBS crystallite size of both blown and BO-85 films exhibited negligible changes. However, *X_c,WAXD_* of BO-PLA6/PBS4-95 and BO-PLA6/PBS4/SA-95 films increased with elevated stretching temperature, indicating the promotion of PBS crystallization under these conditions. Additionally, the <L>_(110)PLA_ values in the PLA-rich BO-PLA8/PBS2-85 and BO-PLA8/PBS2/SA-85 films were greater than those observed in the BO-PLA6/PBS4-85 and BO-PLA6/PBS4/SA-85 films. This suggests that the formation of co-continuous phases or the interconnection of two-phase melts limited PLA crystal growth [11].

#### 3.3.2. Annealing Conditions

In this study, the BO-PLA/PBS and BO-PLA/PBS/SA films were further annealed at selected crystallization temperatures to investigate their microstructural changes and resulting properties. After film biaxial stretching at 85 °C, the BO films were subjected to annealing at various temperatures, including the T_c_ of PBS at 85 °C and the T_cc_ of PBS and PLA at 95 °C and 120 °C, respectively.

**Figure 5:**
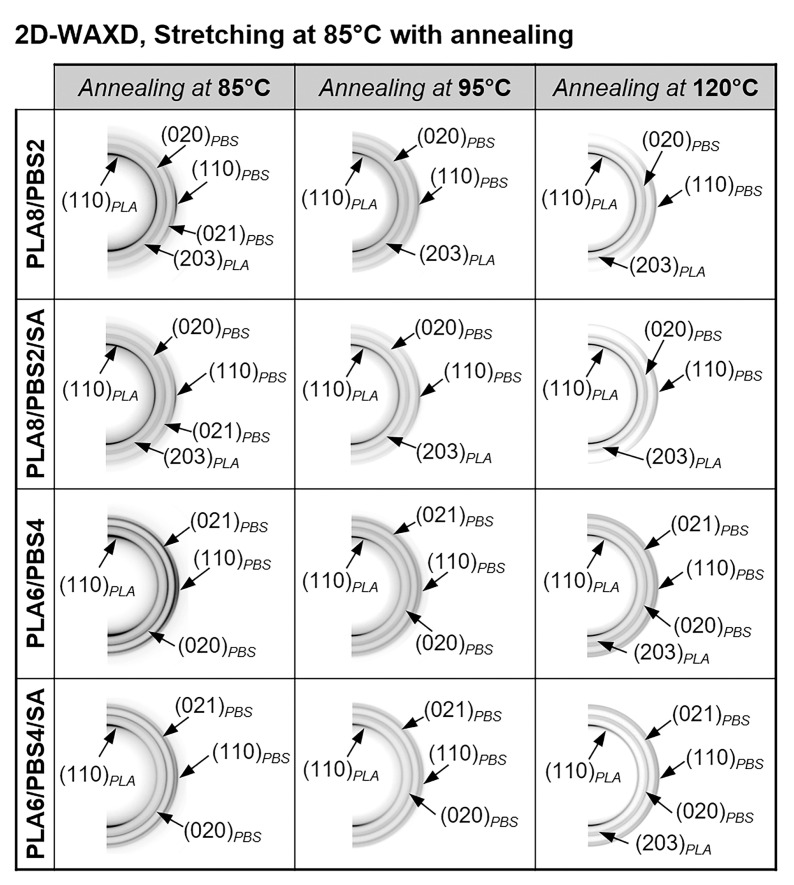
2D-WAXD patterns of BO-PLA/PBS and BO-PLA/PBS/SA films, prepared by stretching at 85 °C and undergoing various annealing temperatures.

Upon annealing the samples at various crystallization temperatures, the 2D-WAXD patterns (Figure 5) remained largely unchanged compared to the non-annealed samples, with the exception of a sharper (203) diffraction peak corresponding to PLA crystals. The PLA phase crystallized in the *δ*-form, as evidenced by the primary diffraction peaks of (110) and (203) planes at 16.2–16.4° and 18.7° 2*θ*, respectively. Additionally, the characteristic diffraction peaks of the *α*-PBS crystals were identified at 19.4° 2*θ* for the (020) plane and 22.5° 2*θ* for the (110) plane. Similarly, the DSC thermograms of the annealed BO-PLA/PBS and BO-PLA/PBS/SA films closely resembled those of non-annealed samples, as shown in Figure 3, with the disappearance of the cold crystallization exotherm and a reduction in the mesophase endotherm (see Figure S2).

**Figure 6:**
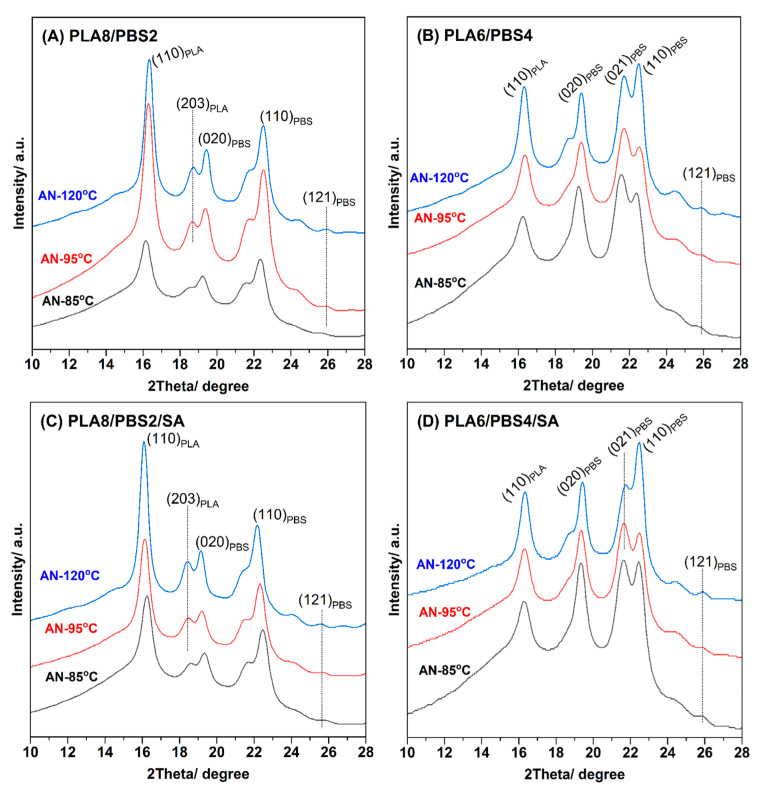
1D-WAXD patterns of BO-PLA/PBS and BO-PLA/PBS/SA films annealed at various temperatures.

As indicated by the 1D-WAXD profiles (Figure 6) and the quantitative data in Table 4, the overall *X_c,WAXD_* in the BO-PLA/PBS-AN85 and BO-PLA/PBS/SA-AN85 films, with both 80/20 and 60/40 weight ratios annealed at 85 °C (the same as the stretching temperature), was higher compared to the non-annealed samples. Furthermore, the overall *X_c,WAXD_* of both BO-PLA/PBS and BO-PLA/PBS/SA films, for both weight ratios, significantly increased, particularly at the higher annealing temperature of 120 °C. Additionally, in the BO-PLA6/PBS4-AN and BO-PLA6/PBS4/SA-AN films with higher PBS content, the (110) and (203) diffraction peaks of PLA crystals sharpened and aligned along the TD as the annealing temperature increased, as observed in Figure 5 and Figure 6. This increase in crystallinity suggests thermal-induced crystallization, wherein the elevated temperatures promoted chain mobility and provided sufficient energy for chains to reorganize into a more ordered crystalline structure, consistent with previous findings [62,63].

When the annealing temperature was increased to T_cc_ of PBS and PLA at 95 °C and 120 °C, not only did the overall *X_c,WAXD_* increase, but the crystallite sizes of PLA phase (<L>_(110)PLA_ and <L>_(203)PLA_ in Table 4) also grew, particularly in the BO-PLA8/PBS2 and BO-PLA8/PBS2/SA films with higher PLA content. Notably, the SA-miscibilized PLA/PBS region did not significantly affect changes in <L>_(110)PLA_ and <L>_(203)PLA_. This suggests that thermal-induced crystallization was the dominant factor in the crystalline PLA phase, rather than a homogeneously blended structure, particularly at elevated temperatures [64,65,66].

Furthermore, as the annealing temperature increased, a similar trend was observed for <L>_(020)PBS_ and <L>_(110)PBS_ of the main PBS diffraction planes, with a preferential alignment along the MD. Although the BO-PLA/PBS and BO-PLA/PBS/SA films were annealed at 120 °C, which exceeds the T_m_ of PBS, the PBS chains were able to recrystallize, likely influenced by the crystalline PLA phase, which remained stable during annealing. This was evidenced by the slightly higher *X_c_* in the BO films annealed at 120 °C, as determined from both DSC thermograms and 1D-WAXD patterns.

#### 3.3.3. Thermal Shrinkage

Thermal shrinkage is a significant challenge for PLA-based films, limiting their application range. This shrinkage occurs because the polymer chains are stretched during film processing, storing conformational energy in the amorphous phase. When heated above T_g_, the stored energy is released, allowing the polymer chains to relax into a more isotropic state, resulting in macroscopic shrinkage. This effect is more prominent in amorphous areas and decreases with increasing crystallinity [67], as demonstrated in our previous work, where the shrinkage of blown PLA films annealed at 100 °C for 60 min was 9.33% in MD and 18% in TD [33].

**Figure 7:**
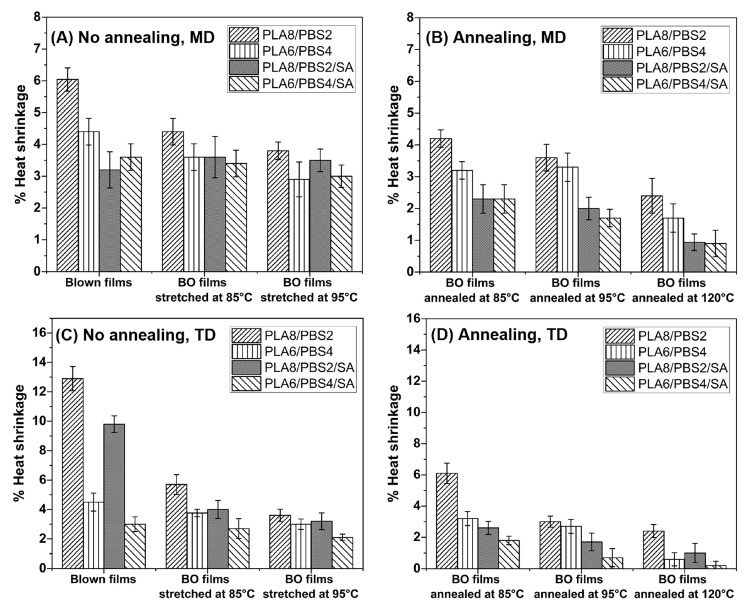
Heat shrinkage percentage of biaxially processed PLA/PBS and PLA/PBS/SA films at varied stretching and annealing temperatures, measured along the MD (**A**,**B**) and TD (**C**,**D**).

In this study, heat shrinkage tests at 120 °C for 60 min (Figure 7) revealed shrinkage values of 6% in MD and 13% in TD for the blown PLA8/PBS2 and PLA8/PBS2/SA films, which have a PLA-rich composition. These values are higher than those observed in blown PLA6/PBS4 and PLA6/PBS4/SA films, which contain a higher proportion of the highly crystalline PBS phase. PBS, known for its excellent thermal resistance, has been shown to significantly increase the softening point of pure PLA from 60–70 °C to above 90 °C when blended, thereby playing a crucial role in reducing thermal shrinkage [14,68].

When the biaxial stretching was applied, the heat shrinkage significantly decreased, particularly in the TD. As shown in Figure 7C, the heat shrinkage of BO-PLA8/PBS2 and BO-PLA8/PBS2/SA films was considerably lower compared to blown PLA8/PBS2 and PLA8/PBS2/SA films. This suggests that the blown films with a PLA-rich phase typically contain many oriented PLA chains in the amorphous regions, as confirmed by the absence of PLA crystal diffraction planes in Figure 4B, which can revert to a random coil configuration when heated above T_g_, resulting in high heat shrinkage percentage [69]. In contrast, biaxial stretching in BO-PLA8/PBS2 and BO-PLA8/PBS2/SA films promoted the complete crystallization of the PLA phase, resulting in isotropic alignment of structures in both MD and TD, which assisted in maintaining film dimensions when exposed to 120 °C. This process facilitated a well-dispersed crystalline phase, significantly enhancing the film dimensional stability. Similar findings were reported by Tsai et al. [70] in their study on BOPLA films. The comparable shrinkage values in both the MD and TD of BO-PLA/PBS and BO-PLA/PBS/SA films indicate more uniform shrinkage between these directions, which can be explained by the role of biaxial stretching in promoting a uniform crystallization of PLA and PBS, thereby reducing shrinkage in all directions.

Moreover, biaxial stretching combined with annealing further promoted the crystallization of both PLA and PBS phases, leading to slightly enhanced the dimensional stability of the annealed films. This observation is consistent with previous studies, which indicated that high-temperature annealing induced chain relaxation, reducing internal stress, as reported by Wu et al. [34]. Additionally, Tsai et al. [70] demonstrated that increasing the annealing temperature from 110 °C to 130 °C significantly raised the *X_c,PLA_* of pure BOPLA films from 18.9% to 34%, resulting in a corresponding reduction in heat shrinkage from 12.5% to 8.5%.

Furthermore, the BO-PLA/PBS/SA films exhibited a slight decrease in shrinkage compared to BO-PLA/PBS films. This decrease can be attributed to the presence of the SA-miscibilized PLA/PBS region, which enhances the thermal stability of the films. The improved miscibility achieved through reactive blending likely contributes to this enhanced thermal stability, underscoring the effectiveness of SA as a compatibilizer in these blends. This performance is comparable to previous reports using various reactive compatibilizers and copolymers, such as isocyanate compounds [24,25], peroxide compounds [19,30,31], epoxy-functional copolymers [23,29], and PLA- or PBS-based copolymers [22,44,48].

### 3.4. Mechanical Properties and Oxygen Permeation of Biaxially Processed PLA/PBS and PLA/PBS/SA Thin Films

#### 3.4.1. Impact Strength

Mechanical strength is a critical property for the practical utilization of polymers, particularly in packaging materials. For instance, PET exhibits an impact strength of 55–60 kJ/m^2^, making it suitable for lid film applications [71]. In contrast, PLA is inherently brittle with a significantly lower impact strength of approximately 1.3 kJ/m^2^, far below that of conventional non-biodegradable plastics [72].

**Figure 8:**
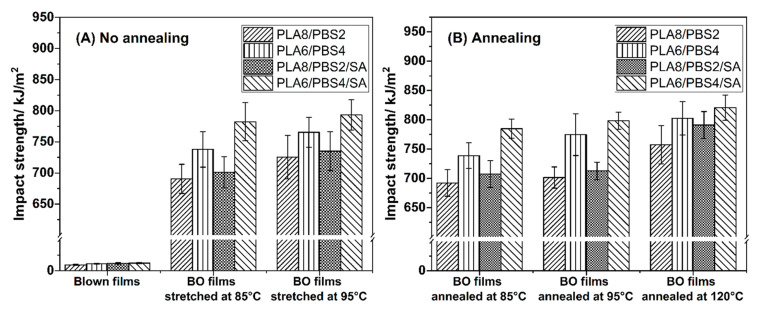
Impact strength of biaxially processed PLA/PBS and PLA/PBS/SA thin films (**A**) without annealing and (**B**) with annealing at different temperatures.

In this study, the impact strength of BO-PLA/PBS and BO-PLA/PBS/SA films was evaluated through puncture resistance testing. Figure 8 illustrates the impact strength of these BO films compared to their blown films, emphasizing the influence of strain-induced crystallization under varying biaxial stretching conditions.

After blending PLA with PBS, the impact strength values of the blown PLA/PBS and PLA/PBS/SA films ranged between 9 kJ/m^2^ and 15 kJ/m^2^, with variations in PBS content. The incorporation of SA did not significantly enhance impact strength. This can be attributed to the high crystallinity of PBS, which promotes the formation of small crystals within the PLA matrix, thus enhancing impact resistance. These findings are consistent with previous reports, which demonstrated improved impact strengths of 8.7 kJ/m^2^ and 10.4 kJ/m^2^ for reactive PLA/PBS 70/30 blends, where glycidyl methacrylate [20] and polyaryl polymethylene isocyanate [24] served as reactive compatibilizers, facilitating cross-linking between PLA and PBS chains. Moreover, SA can be identified as an effective compatibilizer, improving the toughness of the PLA/PBS blend compared to those previous studies.

Notably, the impact strength of BO films stretched at 85 °C without annealing showed a dramatic increase, reaching values between 650 kJ/m^2^ and 750 kJ/m^2^. This substantial improvement highlights the critical role of enhanced PLA crystallization, where larger crystals form through strain-induced crystallization, resulting in highly ordered molecular alignment and uniform crystal dispersion, significantly boosting the material’s impact resistance.

Meanwhile, as the stretching temperature increased, the impact strength of both BO-PLA/PBS and BO-PLA/PBS/SA films showed a moderate increase. This is attributed to the elevated level of strain-induced crystallization at T_cc,PBS_, where crystallization of both PLA and PBS during biaxial stretching was facilitated by enhanced chain mobility at higher temperature. Additionally, the incorporation of SA, which improved miscibility in the PLA/PBS matrix, resulted in slightly higher impact strengths compared to the BO-PLA/PBS films.

When the annealing conditions were applied to BO-PLA/PBS and BO-PLA/PBS/SA films, the impact strength exhibited an increasing trend with rising annealing temperature. This phenomenon corresponds to the growth in crystallite sizes of both the PLA and PBS phases, as well as an increase in overall *X_c_*, which restricts chain movement due to tightly packed structures. When stress is applied to the material, the ordered crystalline regions resist deformation more effectively than the amorphous regions. Additionally, the crystalline regions help distribute and transfer the load across the material, thereby preventing localized deformation [73].

#### 3.4.2. Oxygen Permeation

**Figure 9:**
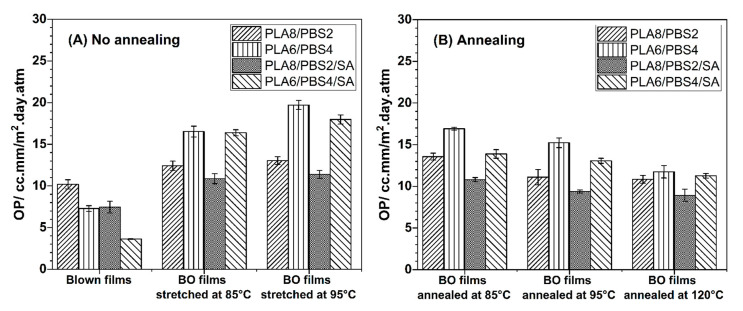
Oxygen permeation of biaxially processed PLA/PBS and PLA/PBS/SA thin films (**A**) without annealing and (**B**) with annealing at different temperatures.

In this study, the oxygen permeabilities of biaxially processed PLA/PBS and PLA/PBS/SA thin films were measured, as shown in Figure 9. To ensure accuracy, the oxygen transmission rate (OTR) was converted to the oxygen permeation coefficient (OP), thereby minimizing the influence of film thickness on the results. Typically, pure PLA film is classified as having moderate oxygen transmission, with values ranging from 40 to 80 cc.mm/m^2^.day.atm [74]. In Figure 9, the OP values of blown PLA8/PBS2 and PLA6/PBS4 films gradually decreased with increasing PBS content. This suggests that the larger <L>_(020)PBS_ and <L>_(110)PBS_ (approximately > 20 nm), along with the isotropic orientation of these PBS crystals, effectively reduced the free volume within the material, thus hindering the diffusion of oxygen molecules. Furthermore, the OP values of the blown PLA8/PBS2/SA and PLA6/PBS4/SA films were lower, indicating that improved interfacial adhesion through the SA-miscibilized region further reduced oxygen transmission.

For BO-PLA/PBS and BO-PLA/PBS/SA films stretched at 85 °C and 95 °C, the OP values tended to increase, despite relatively high overall *X_c,WAXD_* values and a decrease in <L>, as measured by 1D-WAXD patterns. This can be attributed to the anisotropic orientation of PBS crystals along the MD in BO-PLA8/PBS2 and BO-PLA8/PBS2/SA films, combined with the orientation of PLA crystals along the TD in BO-PLA6/PBS4 and BO-PLA6/PBS4/SA films, which likely contributed to the reduced chain entanglement in the amorphous region. This orientation may have also lessened the tortuosity of the gas diffusion pathway, a key factor influencing gas barrier performance [53,75].

Nevertheless, when the BO films were subsequently annealed, particularly at the high temperature of 120 °C, there was a notable reduction in OP values. This can be attributed to the thermal-induced crystallization of PLA and PBS, which, in conjunction with the recrystallization of less ordered PLA and PBS chains, led to an increase in overall *X_c_* and further restricted oxygen permeability. This observation aligns with previous studies, which demonstrated that annealing PLA at 125 °C for more than 20 min achieved *X_c,PLA_* values exceeding 40% [76]. In contrast, the BO films in this study were annealed for a shorter duration, resulting in *X_c,PLA_* values less than 40% and the formation of *δ*-PLA crystals.

## 4. Conclusions

In this study, the SA effectively served as a reactive compatibilizer, enhancing interfacial adhesion between the PLA and PBS phases to form an SA-miscibilized region. A co-continuous phase morphology was observed when the PLA/PBS blend ratio approached 50/50 by weight. Increasing the PBS content promoted PLA crystallization through the nucleating effect of rapidly crystallizable PBS. However, the incorporation of the SA-miscibilized region did not significantly improve PLA crystallization, and slightly interfered with PBS crystallization in the melt blends. WAXD data from the PLA/PBS and PLA/PBS/SA blends and blown films confirmed the dominance of PBS crystalline phase in the *α*-form, with no evidence of a PLA crystalline phase. Strain-induced crystallization through biaxial stretching at both T_c,PBS_ and T_cc,PBS_ facilitated PLA crystallization, especially in PLA-rich blends, rather than blown film extrusion. The combination of biaxial stretching, increased PBS content, and the SA-miscibilized region favored PLA crystal growth in the *δ*-form. Additionally, raising the stretching temperature from 85 °C and 95 °C promoted the crystallization of both PLA and PBS, independent of the increasing PBS content in BO-PLA/PBS and BO-PLA/PBS/SA films. Meanwhile, the annealing conditions in BO films improved the crystallization of PLA and PBS, particularly at the high temperature of T_cc,PLA_. This process intensified the same crystalline forms, resulting in a more stable crystalline phase and larger PLA and PBS crystallite sizes. However, the PBS-dominant crystalline phase in BO-PLA6/PBS4 and BO-PLA6/PBS4/SA films disrupted the alignment of PLA crystals in the MD, in contrast to the PLA-rich BO films, which exhibited isotropic crystal orientation. A comparison between blown and BO films showed that biaxial stretching, particularly when combined with annealing, reduced thermal shrinkage by promoting the crystalline structures of PLA and PBS aligned in both the MD and TD. Moreover, the biaxial-stretching-induced crystallization of PLA and PBS led to a substantial increase in impact strength, with a 70–80-fold improvement, whereas applying annealing, especially at high temperatures, showed only a slight improvement of 10–20% in impact strength, compared to BO films without annealing. Oxygen permeability tests indicated lower transmission in SA-miscibilized blown films due to reduced free volume, while BO films exhibited increased permeability as a result of anisotropic crystal orientation, which reduced chain entanglement and gas diffusion tortuosity. However, annealing BO films at high temperatures (T_cc,PBS_ and T_cc,PLA_) potentially enhanced oxygen barrier properties by promoting PLA and PBS crystallization with more stable crystal structures.

## Figures and Tables

**Table 1:** Summary of sample compositions and BO film preparation conditions.

Sample Code	Composition			BO Film Preparation Conditions	
	PLA (wt.%)	PBS (wt.%)	SA (phr)	Stretching Temperature (°C)	Annealing Temperature (°C)
PLA8/PBS2	80	20	-	-	-
PLA7/PBS3	70	30	-	-	-
PLA6/PBS4	60	40	-	-	-
PLA8/PBS2/SA	80	20	5	-	-
PLA7/PBS3/SA	70	30	5	-	-
PLA6/PBS4/SA	60	40	5	-	-
BO-PLA8/PBS2-85	80	20	-	85	-
BO-PLA6/PBS4-85	60	40	-	85	-
BO-PLA8/PBS2/SA-85	80	20	5	85	-
BO-PLA6/PBS4/SA-85	60	40	5	85	-
BO-PLA8/PBS2-95	80	20	-	95	-
BO-PLA6/PBS4-95	60	40	-	95	-
BO-PLA8/PBS2/SA-95	80	20	5	95	-
BO-PLA6/PBS4/SA-95	60	40	5	95	-
BO-PLA8/PBS2-AN85	80	20	-	85	85
BO-PLA6/PBS4-AN85	60	40	-	85	85
BO-PLA8/PBS2/SA-AN85	80	20	5	85	85
BO-PLA6/PBS4/SA-AN85	60	40	5	85	85
BO-PLA8/PBS2-AN95	80	20	-	85	95
BO-PLA6/PBS4-AN95	60	40	-	85	95
BO-PLA8/PBS2/SA-AN95	80	20	5	85	95
BO-PLA6/PBS4/SA-AN95	60	40	5	85	95
BO-PLA8/PBS2-AN120	80	20	-	85	120
BO-PLA6/PBS4-AN120	60	40	-	85	120
BO-PLA8/PBS2/SA-AN120	80	20	5	85	120
BO-PLA6/PBS4/SA-AN120	60	40	5	85	120

**Table 2:** Degree of crystallinity and crystallite sizes of PLA/PBS and PLA/PBS/SA blends.

Sample Code	DSC Thermograms		1D-WAXD Patterns			
	*X_c,PBS_* (%)	*X_c,PLA_* (%)	*X_c,WAXD_* (%)	<L>_(020)PBS_ (nm)	<L>_(021)PBS_ (nm)	<L>_(110)PBS_ (nm)
PLA8/PBS2	40.4	7.7	6.3	18.4	-	14.9
PLA7/PBS3	40.7	9.0	7.9	16.0	6.2	17.9
PLA6/PBS4	40.6	13.3	15.9	22.5	11.3	18.1
PLA8/PBS2/SA	36.8	7.9	7.6	17.2	-	20.6
PLA6/PBS4/SA	39.4	12.2	17.9	21.0	7.6	22.4

**Table 3:** Degree of crystallinity and crystallite size of blown and BO films without annealing for PLA/PBS and PLA/PBS/SA blends.

Sample	DSC Thermograms		1D-WAXD Patterns					
	*X_c,PBS_* (%)	*X_c,PLA_* (%)	*X_c,WAXD_* (%)	<L>_(110)PLA_ (nm)	<L>_(203)PLA_ (nm)	<L>_(020)PBS_ (nm)	<L>_(021)PBS_ (nm)	<L>_(110)PBS_ (nm)
blown PLA8/PBS2	14.9	20.1	7.4	-	-	20.2	6.7	23.1
BO-PLA8/PBS2-85	50.1	25.2	18.4	13.0	14.0	17.6	15.0	15.4
BO-PLA8/PBS2-95	59.5	26.8	26.3	19.4	25.5	19.7	13.3	15.1
blown PLA8/PBS2/SA	23.6	20.2	7.8	-	-	23.0	11.7	18.2
BO-PLA8/PBS2/SA-85	43.2	32.1	18.8	12.6	13.5	18.1	12.7	13.0
BO-PLA8/PBS2/SA-95	59.4	34.1	29.1	19.8	19.2	16.7	15.4	18.5
blown PLA6/PBS4	28.6	24.0	18.7	-	-	24.7	13.5	15.8
BO-PLA6/PBS4-85	54.3	31.4	17.7	9.0	-	15.1	12.0	15.9
BO-PLA6/PBS4-95	58.4	30.2	24.1	17.8	4.4	15.2	11.8	16.4
blown PLA6/PBS4/SA	30.7	23.4	23.6	-	-	19.7	6.7	23.1
BO-PLA6/PBS4/SA-85	55.5	30.1	20.6	8.7	-	16.3	11.6	15.7
BO-PLA6/PBS4/SA-95	63.6	34.7	25.3	18.1	5.7	16.3	13.4	17.6

**Table 4:** Degree of crystallinity and crystallite size of BO-PLA/PBS and BO-PLA/PBS/SA films after annealing at varied temperatures of 85 °C, 95 °C, and 120 °C.

Sample	DSC Thermograms		1D-WAXD Patterns					
	*X_c,PBS_* (%)	*X_c,PLA_* (%)	*X_c,WAXD_* (%)	<L>_(110)PLA_ (nm)	<L>_(203)PLA_ (nm)	<L>_(020)PBS_ (nm)	<L>_(021)PBS_ (nm)	<L>_(110)PBS_ (nm)
BO-PLA8/PBS2-AN85	39.7	21.5	21.0	14.4	14.9	17.4	15.2	15.4
BO-PLA8/PBS2-AN95	54.3	27.1	25.5	17.7	16.0	20.7	15.6	16.5
BO-PLA8/PBS2-AN120	59.0	29.4	31.6	20.0	16.9	24.1	15.6	18.8
BO-PLA8/PBS2/SA-AN85	59.3	30.6	22.9	15.5	15.0	16.4	13.3	14.0
BO-PLA8/PBS2/SA-AN95	57.0	31.2	27.0	17.2	16.4	18.8	16.2	16.8
BO-PLA8/PBS2/SA-AN120	52.6	31.2	32.9	20.7	17.8	23.7	15.8	19.1
BO-PLA6/PBS4-AN85	62.0	30.1	20.4	12.6	-	14.0	12.3	15.7
BO-PLA6/PBS4-AN95	62.2	30.3	22.5	15.9	5.0	18.8	12.9	16.0
BO-PLA6/PBS4-AN120	64.4	31.0	28.6	17.6	9.4	23.5	14.1	19.4
BO-PLA6/PBS4/SA-AN85	57.8	29.6	21.5	13.8	-	14.5	12.8	16.6
BO-PLA6/PBS4/SA-AN95	66.8	35.1	23.3	14.7	6.9	23.3	13.4	17.5
BO-PLA6/PBS4/SA-AN120	61.7	33.6	32.9	16.9	8.3	31.7	11.1	22.1

## Data Availability

The original contributions presented in the study are included in the article/supplementary material, further inquiries can be directed to the corresponding author.

## Supplementary Materials

The following supporting information can be downloaded at: https://www.mdpi.com/article/10.3390/polym16213033/s1. Figure S1. Cooling stage DSC thermograms for PLA/PBS and PLA/PBS/SA blends with weight ratios of 80/20 and 60/40. Figure S2. First heating DSC thermograms of BO-PLA/PBS and BO-PLA/PBS/SA films under varied annealing conditions.
