# Supplementary material for: Enhanced Impact Resistance, Oxygen Barrier, and Thermal Dimensional Stability of Biaxially Processed Miscible Poly(Lactic Acid)/Poly(Butylene Succinate) Thin Films
Source: Polymers (Basel). 2024 Oct 29;16(21):3033. doi: 10.3390/polym16213033 (PMC11548280; doi:10.3390/polym16213033)
Supplement: Supplementary file 1 [file polymers-16-03033-s001.zip › polymers-3269653-supplementary.pdf]

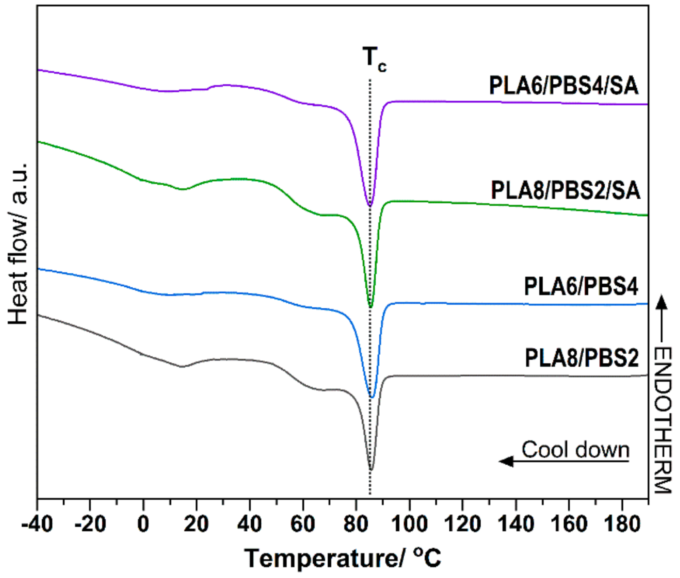

Figure S1. Cooling stage DSC thermograms for PLA/PBS and PLA/PBS/SA blends with weight ratios of 80/20 and 60/40.

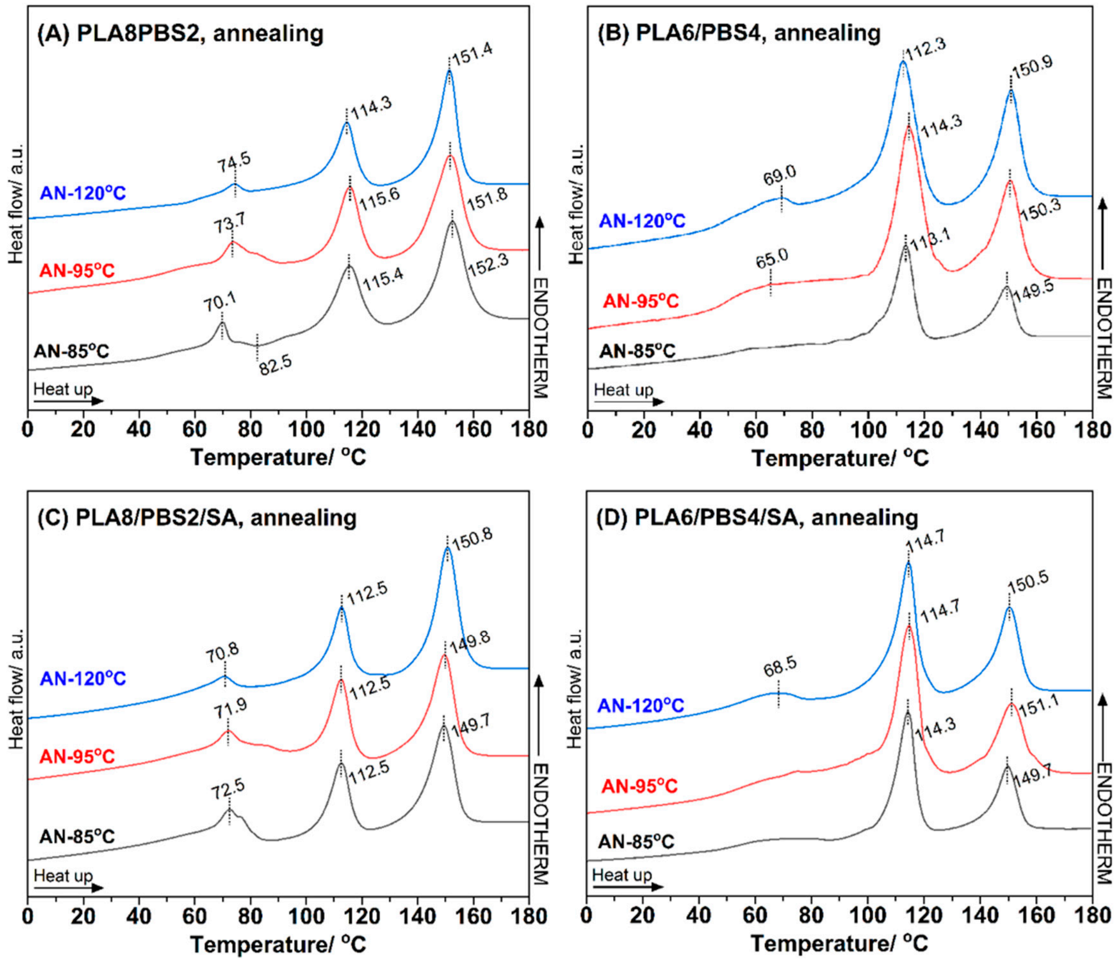

Figure S2. First heating DSC thermograms of BO-PLA/PBS and BO-PLA/PBS/SA films under varied annealing conditions.
